# Comparison of hemodynamic changes and prognosis between stenting and standardized medical treatment in patients with symptomatic moderate to severe vertebral artery origin stenosis

**DOI:** 10.1097/MD.0000000000014899

**Published:** 2019-03-15

**Authors:** Jia-Xin Shao, Yun-Ao Ling, Hua-Ping Du, Guo-Jie Zhai, Yuan Xu, Yong-Jun Cao

**Affiliations:** a Department of Neurology, the First People Hospital of Wujiang District, 169 Gong-Yuan Road, Wujiang District, Suzhou 215200; b Department of Neurology, The Second Affiliated Hospital of Soochow University, No. 1055, Sanxiang Road, Suzhou, Jiangsu 215000, China.

**Keywords:** efficacy, standardized medical treatment, stenting, transcranial Doppler, vertebral artery stenosis

## Abstract

This study aimed to compare the clinical efficacy of stenting compared with standardized medical treatment in patients with moderate to severe vertebral artery origin stenosis (VAOS).

Patients diagnosed with moderate to severe VAOS and indicated to undergo vertebral artery stenting were enrolled. Patients were divided into stenting group and standardized medical treatment group. All patients underwent transcranial Doppler (TCD) before and after treatment. Incidence of new cerebral infarction, transient ischemic attack (TIA), improvement of clinical symptoms, and National Institutes of Health Stroke Scale (NIHSS) score were observed.

A total of 98 patients were enrolled. Vertebral artery stenting implant was accepted by 43 patients. Two weeks after treatment, the NIHSS score in the stenting group decreased significantly compared to that in the standardized medical treatment group. The modified Rankin Scale (mRS) score in the stenting group at three months was significantly lower than that in the medical treatment group (*P* = .044). The extent of vascular stenosis in the stent group decreased significantly (76.5 ± 10.0% vs. 13.7 ± 5.9%, *t* = 35.878, *P* = .000). The adverse events occurred in 9 (16.4%) patients in the medical treatment group and 5 (11.6%) in the stenting group (*P* = .506). There was one case with new cerebral infarction in the stenting group, whereas the medical treatment group showed 1 case with TIA and three with new cerebral infarction during follow-up after 3 months. The peak systolic velocity (PSV), end diastolic velocity (EDV), pulsatility index (PI) of stenosis vertebral artery, and PSV of basilar artery were significantly higher in the stent group than those in the standardized medical group (*P* < .05).

Stenting for VAOS, rather than standardized medical treatment, can effectively relieve vascular stenosis, alter vertebral-basilar artery hemodynamics, and improve neurological function, with low perioperative complications.

## 1 Introduction

Vertebral artery origin stenosis (VAOS) has received much attention in the recent years as a main reason for stroke in the posterior circulation. Data from the Oxford Vascular Study and New England Registry showed that 26 to 33% patients with posterior stroke have vertebral artery stenosis or occlusion.^[1,2]^

Despite medical treatment in patients with symptomatic vertebral artery stenosis, the risk of recurrent stroke remains 25% at 90 days.^[3]^ Endovascular treatment of symptomatic vertebral artery stenosis with percutaneous transluminal angioplasty (PTA) and stenting has been introduced as a promising option and is widely used in clinical practice.^[4]^ The Vertebral Artery Ischaemia Stenting Trial showed that stenting in extracranial stenosis appears safe with low rate of complications.^[5]^ However, there is a paucity of evidence showing that stenting is effective for improvement in blood flow and prognosis.

Thus, we compared the major periprocedural vascular complications, hemodynamics, and prognosis of patients who underwent stenting and those who underwent standardized medical treatment for symptomatic vertebral artery stenosis.

## 2 Methods

### 2.1 Study design and participants

Our study was a single center, prospective, non-randomized controlled study. Of the patients with posterior circulation TIA or stroke form the First People Hospital of Wujiang District from January 2016 to July 2017. Ninety-eight participants aged 18 years or older with VAO S ≥50% as assessed by the digital subtraction angiography (DSA) that reached the standards of stent treatment as previously reported were included in the study.^[6]^ The degree of stenosis was calculated by a formula: (1 − *N*/*D*)∗100%.^[7]^*N* represents the residual diameter of a point distal to the stenosis and *D* represents the normal diameter. The exclusion criteria were: (1) patients with mRS >3 or severe illness, like heart failure or advanced cancer; (2) intracranial vascular malformation or aneurysm; (3) VAOS caused by dissection or vasculitis; (4) patients with dementia or other mental illness. Patients were assigned by their willingness to the stenting or to standardized medical treatment groups. All participants provided written informed consent. This study was approved by the Ethical Committee of the First People Hospital of Wujiang District.

### 2.2 Standardized medical treatment

Fifty-five patients in the drug group received standardized medical treatment including antiplatelets agents, statins, and control of vascular risk factors as previous reported.^[7]^

### 2.3 VA stenting

Forty-three patients in the stent group received aspirin 100 mg per day and clopidogrel 75 mg per day for at least three days before stent implanting. The procedure was typically performed by femoral access, and a 6F guiding catheter was placed. Heparin was administered to maintain the coagulation time and avoid thrombosis. Before operation, the location and extent of VA stenosis was reconfirmed. A 0.014-inch microwire was used to cross the stenosis. Thereafter acrossing the stenosis, an angioplasty balloon was exchanged for predilation of the stenosis, and a stent was deployed. Post-dilation was allowed if residual stenosis existed after the operation. A degree of stenosis <30% is indicative of the success of the operation. All operations were performed by the same interventionist. After the procedure, aspirin and clopidogrel were continued for at least 3 months. Thereafter, aspirin was continued indefinitely.

### 2.4 Follow-up

Data of demographic characteristics, risk factors, medical history, location of stroke, and degree of stenosis were collected. Clinical follow-ups were performed at 2 weeks and 3 months after the operation by a trained experienced neurologist. The follow-up date included the National Institutes of Health Stroke Scale (NIHSS) and modified Rankin Scale (mRS) scores, transcranial Doppler, periprocedural complications and posterior circulation stroke or transient ischemic attack (TIA). Patients who underwent stent placement were assessed using MR angiography or CT angiography to evaluate the in-stent restenosis 3 months after treatment. Restenosis was defined as residual stenosis reaching at least 50% or occlusion.^[8]^

### 2.5 Statistical analysis

SPSS 19.0 was used for data analysis. Continuous variables were presented as mean and standard deviation; they were compared using the Student *t*-test, and ANOVA was used for multiple comparisons. SNK-q test was used for comparison between the groups. The categorical variables were presented as frequencies and percentages, and they were compared using the χ^2^ test or Fisher exact test. *P* < .05 was considered statistically significant.

## 3 Results

### 3.1 Baseline characteristics

Of the 98 patients enrolled, 55 were assigned to the standardized medical treatment group and 43 to the stent group. The baseline characteristics, risk factors, medical history, location of stroke, NIHSS score, mRS score, degree of stenosis, and other clinical data of participants in this study were well matched (Table 1).

**Table 1:**
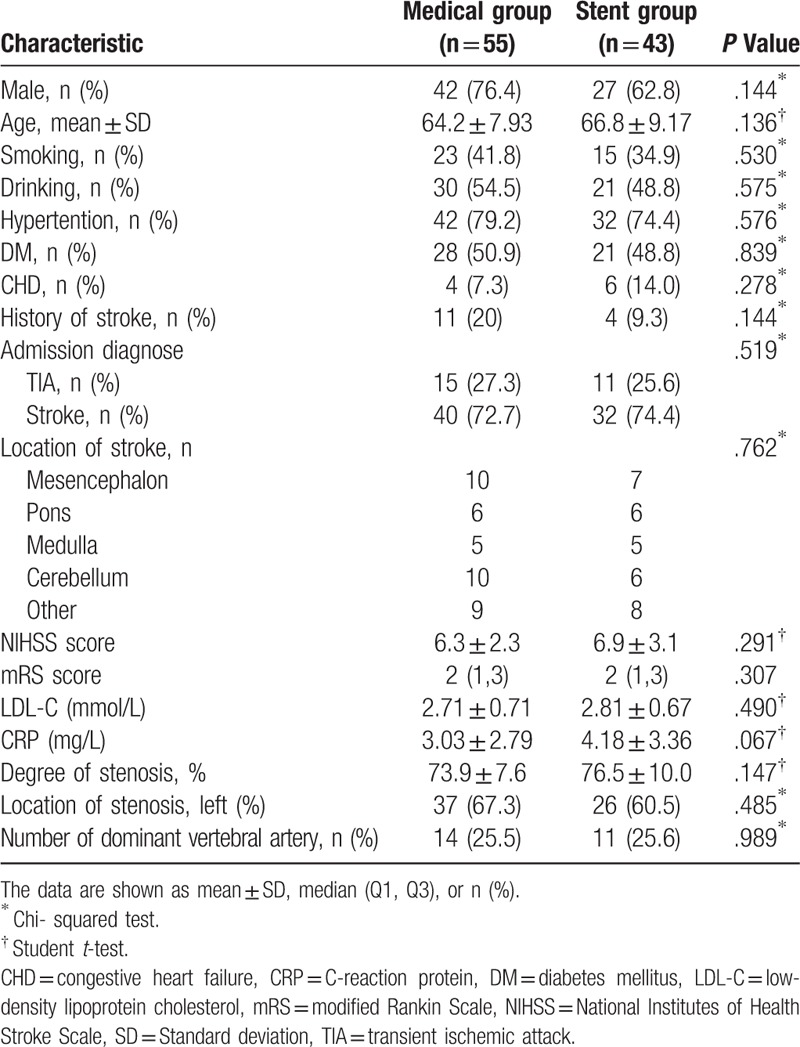
Baseline characteristics of study participants.

### 3.2 Comparison of the clinical curative effects between two groups

There was no significant difference in NIHSS score at admission between the two groups. Two weeks after treatment, the NIHSS score in the medical treatment and stent groups decreased by 1.9 ± 0.7 and 2.6 ± 1.3 respectively (*P* = .002). Three months after treatment, the median mRS score in the stent group was lower than that in medical group, respectively (1(IQR = 1–3) vs 2 (IQR = 1–2), *P* = .044). In the stent group, the admission average stenosis at admission was 76.5% ± 10.0%, which decreased significantly to 13.7% ± 5.9% (*P* = .000) after treatment (Table 2).

**Table 2:**
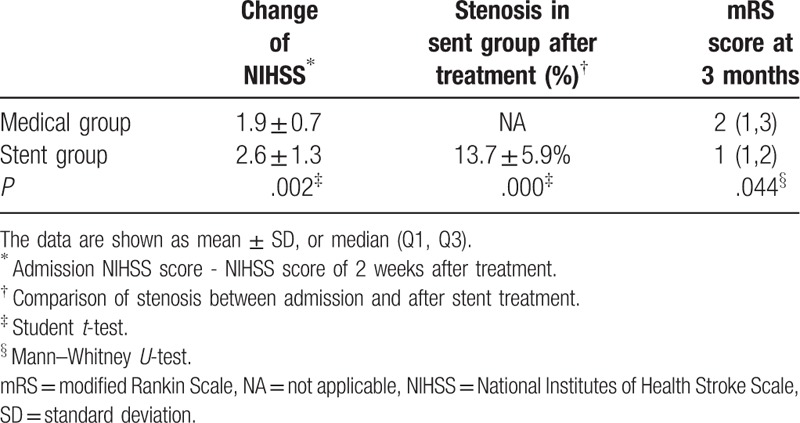
clinical curative effects of two groups.

### 3.3 Safety and outcome between medical and stent treatment

No cerebral hyperperfusion syndrome, cerebral vasospasm, intracerebral hemorrhage (ICH), arterial dissection, and epilepsia occurred during the stenting procedure. During the follow-up, only 1 (2.3%) patient had recurrent nonfatal posterior circulation stroke in the stent group, and 4 (7.3%) patients had ischemic events, including three cases with stroke and 1 with TIA (*P* = .381). No patient died until 3 months after treatment. Three (5.5%) patients in the medical group and 3 (7.0%) patients in the stent group had gastrointestinal bleeding. Hepatic insufficiency occurred in 2 (3.6%) patients in the medical group and 1 (2.3%) patient in the stent group with 3 months. The total adverse events occurred in 9 (16.4%) patients in the medical group and 5 (11.6%) in the stent group, and there was no statistically significant difference between the 2 groups (*P* = .506). No restenosis occurred in the stent group 3 months after stent treatment (Table 3).

**Table 3:**
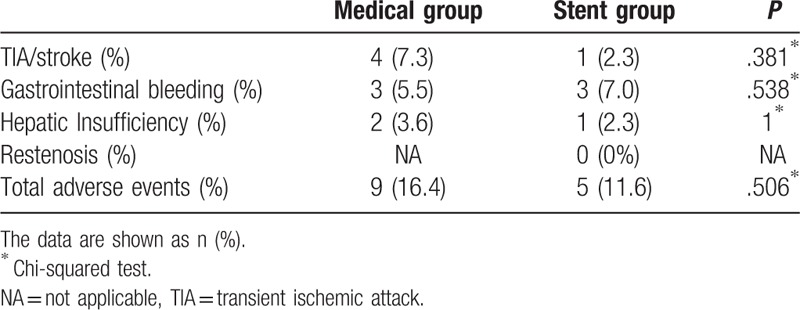
Adverse events.

### 3.4 Hemodynamics of the vertebrobasilar artery of in the medical treatment and stent groups

All patients were administered transcranial Doppler (TCD) 2 weeks and 3 months after treatment. There were no differences in the peak systolic velocity (PSV), end diastolic velocity (EDV), and pulsatility index (PI) of the intracranial vertebral artery on the affected side and the basilar artery (BA) between the medical and stent groups before treatment. In the normal intracranial vertebral artery, the hemodynamics showed no significant differences between the 2 groups. Two weeks and 3 months after treatment, PSV, EDV, and PI of the intracranial vertebral artery of affected side in the stent group were higher than those in the medical treatment group. The PSV, EDV, and PI of the normal aside in the stent group showed no significant changes compared to those of the medical treatment group. The PSV and PI of the basilar artery in the medical group were lower than those in the stent group (Table 4).

**Table 4:**
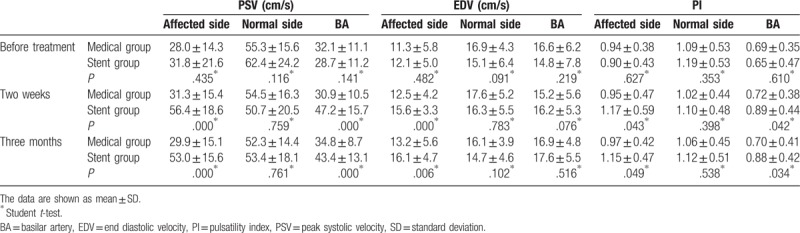
Hemodynamics of intracranial vertebrobasilar artery between two groups.

In the medical group, there were no changes in hemodynamics after treatment. However, the PSV and mean velocity of the intracranial vertebral artery of affected side and basilar artery were increased significantly 2 weeks and 3 months after stent treatment. The EDV of the intracranial vertebral artery of affected side was decreased 2 weeks and 3 months after stent treatment. The PI of the intracranial vertebral artery and basilar artery of affected aside were significantly increased two weeks and three months after stent treatment (Table 5).

**Table 5:**
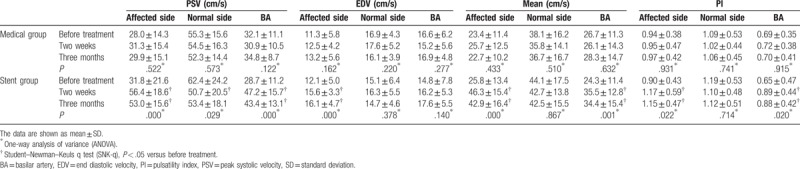
Hemodynamics of intracranial vertebrobasilar artery pre-post treatment.

## 4 Discussion

With the aging of the population and the rapid improvement in economic levels, the incidence of ischemic cerebrovascular disease based on atherosclerosis gradually increased.^[9]^ Wang et al found that the hospital stay was significantly prolonged and the risk of stroke recurrence was significantly higher in stroke patients with atherosclerosis of the head and neck.^[10]^

Vertebral artery stenosis is the main cause of posterior circulation stroke.^[11]^ The patients with moderate and severe stenosis had higher risk of stroke recurrence despite treatment with standardized drugs.^[12]^ With the development of endovascular therapy, endovascular stents have become an important treatment for patients with vertebral artery stenosis; and its safety and effectiveness have been confirmed by many studies. The study by Jenkins et al suggested that the success rate of vertebral artery stenting was 100%, the clinical success rate was over 90%, and the degree of vertebral artery stenosis decreased from 89.2% to 1.4%.^[13]^ The CAVATAS study compared the best drug treatment with endovascular treatment and that with drugs, and found no intracerebral stroke and death in the endovascular treatment group within 30 days, the endpoint events in 2 groups showed no significant difference. The results suggested that endovascular treatment is safety.^[14]^ In our study, the success rate of VOAS was 100%. There were no complications, such as perioperative stroke, cerebral hyperperfusion syndrome, and cerebral vasospasm. The rate of VAOS decreased from 76.5 ± 10.0% to 13.7 ± 5.9%. Some studies showed high preoperative complications. The complications of SAMMPRIS study reached 14.7%, far higher than that with medical treatment.^[15]^ Strict preoperative preparation, standard endovascular treatment, and experienced operator intervention can reduce severe periprocedural complications.

At present, the effect of endovascular treatment and standard drug treatment for vertebral artery stenosis and standard drug treatment is controversial. A study compared the efficacy of stent and drug therapy for symptomatic vertebral artery stenosis.^[7]^ During the follow-up period, seven patients in the drug group and eight in the stent group suffered from a stroke event. There was no significant difference between the 2 groups. The study included patients with intracranial vertebral artery stenosis, and therefore, could not evaluate the difference between the 2 treatments. The CAVATAS study showed that after an average of 4.7 years of random visits, the endovascular treatment group did not show a clear advantage compared with the standard drug treatment; however, the study included a smaller sample size.^[14]^ A recent study showed that after a mean follow-up of 3.5 years, the number of cases of stroke in the stent group and the drug group was 5 and 12, respectively (*P* = .08).^[5]^ However, the randomization time in the drug group was significantly longer than that of in the stent treatment group. Adjusting the final event to a random time, corresponding Hazard Ratio (HR) for primary endpoint was 0.34 (*P* = .046) and showed no significant difference in NIHSS scores between the pre-treatment and the drug treatment group. In our study, after 2-weeks treatment, the NIHSS score in the stent group was significantly lower than that of the drug treatment group; and after 3-months treatment, the mRS score of the stent group was significantly decreased, which suggested that stent treatment can improve the neurological function of the stroke patients compared with drug therapy. In the stent group, there was one case (2.3%) of posterior circulation stroke during follow-up, and four cases of ischemic events (7.3%) in the drug treatment group, and there was no significant difference between the two groups. Considering the short follow-up time and the small number of patients involved, it was necessary to extend the follow-up time and increase the number of patients included.

Vascular ultrasound, the preferred method for patients, is a non-invasive, reproducible, affordable.^[16]^ A number of studies have showed that changes in the distal stenosis, such as reduced flow velocity and decreased pulsatility can be used to evaluate vertebral artery stenosis.^[17,18]^ Studies have shown that in patients with unilateral vertebral artery stenosis, the blood flow velocity of the contralateral vertebral artery is significantly high. The researchers believe that this is due to compensation by the contralateral vertebral artery to ensure normal cerebral perfusion.^[19]^ Our study compared the effect of two different treatment methods on the hemodynamics of the vertebral-basal artery. The results showed that the PSV, EDV in the stenotic vertebral artery and PSV in the basilar artery were significant higher in the stenting group than in the drug treatment group. The results were similar to those of previous studies.

Vertebral stent restenosis is the most important issue in the posterior circulation stent therapy, and it is also a crucial factor that limits the vertebral artery stenting. Studies have shown that the restenosis rate of the vertebral artery in the initial stenting ranged from 30% to 40%, whereas restenosis mostly occurred within the first year after stenting and was pronounced from 6 to 12 months after treatment.^[20]^ In our study, a total of 43 patients underwent stenting of the vertebral artery. The followed-up time was 3 months, and restenosis did not appear. Our study had some limitations. The following factors may have affected the result: first, follow-up time was short; second, the sensitivity of vascular ultrasound is lower than that of DSA. Further research is needed to validate the results of our study.

## 5 Conclusion

Stenting for VAOS, rather than standardized medical treatment, can effectively relieve vascular stenosis, alter vertebral-basilar artery hemodynamics, and improve neurological function, with low perioperative complications.

## Author contributions

**Conceptualization:** Jia-Xin Shao, Yun-Ao Ling, Hua-Ping Du, Yuan Xu, Yong-Jun Cao.

**Data curation:** Jia-Xin Shao, Yun-Ao Ling, Hua-Ping Du, Guo-Jie Zhai.

**Formal analysis:** Jia-Xin Shao, Yun-Ao Ling, Hua-Ping Du, Yuan Xu, Yong-Jun Cao.

**Investigation:** Jia-Xin Shao, Yun-Ao Ling, Hua-Ping Du, Guo-Jie Zhai.

**Methodology:** Yun-Ao Ling.

**Project administration:** Yun-Ao Ling, Yuan Xu, Yong-Jun Cao.

**Resources:** Jia-Xin Shao.

**Supervision:** Yuan Xu, Yong-Jun Cao.

**Validation:** Yun-Ao Ling, Yuan Xu, Yong-Jun Cao.

**Visualization:** Yun-Ao Ling, Hua-Ping Du, Yuan Xu.

**Writing – original draft:** Jia-Xin Shao.

**Writing – review & editing:** Yuan Xu, Yong-Jun Cao.
